# Box–Behnken design based statistical modeling for the extraction and physicochemical properties of pectin from sunflower heads and the comparison with commercial low-methoxyl pectin

**DOI:** 10.1038/s41598-020-60339-1

**Published:** 2020-02-27

**Authors:** Xiaoxia Peng, Guang Yang, Yun Shi, Yifa Zhou, Mengshan Zhang, Shanshan Li

**Affiliations:** 1 Institute of Special Animal and Plant Sciences, Chinese Academy of Agriculture Sciences, Changchun, 130112 China; 2 Jilin Province Key Laboratory on Chemistry and Biology of Natural Drugs in Changbai Mountain, School of Life Sciences, Northeast Normal University, Changchun, 130024 China; 3 Shanxi University of Chinese medicine, Taiyuan, 030024 China

**Keywords:** Biochemistry, Carbohydrates, Polysaccharides

## Abstract

A natural low-methoxyl pectin (LAHP), was extracted with oxalic acid solution from dried heads of sunflower (*Helianthus annuus* L.). The single-factor experiments and response surface methodology (RSM) were used to optimize LAHP extraction conditions. The extraction yield of LAHP was 18.83 ± 0.21%, and the uronic acid content was 85.43 ± 2.9% obtained under the optimized conditions (temperature of 96 °C, time of 1.64 h, oxalic acid concentration of 0.21%). Experimentally obtained values were in agreement with those predicted by RSM model, indicating suitability of the employed model and the success of RSM in optimizing the extraction conditions. LAHP has been characterized by ash content, degree of esterification (DE), galacturonic acid (GalA) content, molecular weight and intrinsic viscosity meanwhile commercial low-methoxyl pectin (CLMP) as comparison. This study finds out a potential source of natural LMP which expands the application scope of sunflower heads. It is an efficient reuse of waste resources and provides a novel thought to explore the natural resources for food and pharmaceutical applications.

## Introduction

Pectin is a kind of complex polysaccharides from plant primary cell walls, always consisting of methyl esterified D-galacturonic acid^1^. Pectin has been widely used in food, pharmacy and cosmetics industries as thickeners, stabilizers, gelling agents^2^ and ingredients of medicine for the treatment of gastroenterological diseases, diabetes, hypertension and cancer^3^.

Up to now, most of commercial pectins produced from citrus peel, apple pomace^4^, sugar beet pulp^5,6^, orange^7^, lemon^8^, banana^9^, peach^10^, durian rind^11^ and soy hulls^12^ were high-methoxyl pectin (HMP, degree of esterification [DE] > 50%)^13^. DE is a primary factor in terms of gelling forming conditions and mechanical properties. HMP usually cannot meet people’s demand on food flavor because of its free of good taste. The gel forming of HMP is governed by hydrogen bonding and hydrophobic interactions, which requires the sugar solution at concentration higher than 55% and pH lower than 3.5. Nevertheless low-methoxyl pectin (LMP)^14^ not only improves taste but also form gel easily induced by the presence of divalent ion, such as Ca^2+^. The ‘egg box’ model^15^ has been widely accepted for calcium pectate gels, which describes the junction zones in LMP chains^16^. LMP could be widely used in low calorie foods or sugar-free food, healthy care medicines and cosmetics industries^17^. In this case, HMP is usually served to manufacture LMP by chemical or enzymatic treatment to hydrolysis of the methyl esters from galacturonic acid C-6 carboxyl groups. However, this kind of synthesized LMP is costly and has hazardous residuals brought in by chemical and enzymatic treatment.

Sunflower (*Helianthus annuus* L.) heads are abundant with natural LMP^18,19^. Although a number of studies on sunflower heads pectin have been reported^20^, it rarely focus on whether the yields, ash content and GalA content are suitable for industrial utilization. It is necessary to optimize the extraction process and conditions to improve the quantity and quality of sunflower heads pectin in order to meet the commercial demands for LMP.

In this paper, single-factor experiments and response surface methodology (RSM) based on a Box-Behnken design (BBD), two most commonly experimental design methods were used to optimize the extraction conditions of pectin, which named as LAHP from sunflower heads. The chemical composition and physical properties of LAHP were also investigated, and then compared with commercial low-methoxyl pectin (CLMP) and the national standards of P.R.C (GB25533-2010). The objective of this study was to find out an abundant source of natural LMP and expands the application scope of sunflower heads. It is an efficient reuse of waste resources and also furnishes a novel thought to explore the natural resources.

## Materials and Methods

### Raw materials and regents

The sunflower heads were cultivated in Baicheng city (Jilin Province, China). CLMP were produced by Yantai Andre Pectin Co. Ltd (5630-02) from the apple pomace. Standard polygalacturonic acid was obtained from Sigma (St. Louis, USA). D-rhamnose, L-arabinose, D-xylose, D-mannose, D-glucose, D-galactose were provided by Sigma (St. Louis, USA). Oxalic acid dihydrate was purchased from Beijing Chemical Works. All reagents used for analytical procedures were analytical grade.

### Extraction of pectin

Dried sunflower head (10.0 g) was soaked in 80% ethanol for 3 h to remove some colored materials, oligosaccharides and other small molecule materials. The pretreated sunflower head was transferred to a 500 mL glass flask placed in an incubator and added 200 mL distilled water. Oxalic acid was added to obtain extraction solvents range from 0.1% to 0.3% concentration and the flask was subsequently incubated with agitation at temperature range from 70 to 100 °C for 1 h to 2 h. The resulting slurries were cooled to room temperature and filtered through gauze of four sheets. The residues were extracted again under the same conditions. The filtrates were combined and centrifuged for 15 min at 4000 rpm to remove solid particles. Three volumes of 95% (v/v) ethanol were added to each extracts in order to precipitate the pectin. Then, the precipitates were collected by centrifugation (4000 rpm/min, 15 min) and re-dissolved in distilled water and lyophilized to obtain sunflower heads pectin (LAHP). The extraction yield (*Y*) was calculated from the following equation^21^.

1 $$Y( \% ,\,w/w)=({m}_{o}/m)\times 100$$

m_o_: weight of extracted pectin, m: weight of dried sunflower heads.

### Experimental design and statistical analysis

RSM is a compilation of statistical and mathematical techniques that are established on the fit of polynomial equation to the experimental data. According to the preliminary range of extraction variables determined by single-factor experiment, a three-level-three-factor BBD was applied to determine the best combination of extraction variables for the production of pectin from sunflower heads. Extraction temperature (X_1_), extraction time (X_2_) and oxalic acid concentration (X_3_) were the independent variables selected to be in this experimental design, the extraction yield and uronic acid content were selected as the responses for the combination of the independent variables. Table 1 lists BBD matrix and the response values carried out for developing the models. Three experiments of each condition were performed and the mean values were stated as observed responses. In order to minimize the effect of unexplained variability, all the experiments were carried out in random order.

**Table 1:** Box-Behnken experimental design and response values for extraction yield and uronic acid content of LAHP.

Run	X_1_^a^	X_2_^b^	X_3_^c^	Yield (%)	Uronic acid content (%)
1	0	−1	1	14.23	66.51
2	1	0	−1	17.28	70.27
3	−1	0	1	10.74	59.62
4	−1	0	−1	10.31	57.09
5	0	0	0	16.46	83.86
6	−1	−1	0	9.42	61.13
7	0	0	0	16.82	85.63
8	0	0	0	16.65	85.02
9	−1	1	0	11.39	67.08
10	0	−1	−1	13.02	61.44
11	0	1	−1	15.37	69.77
12	1	0	1	18.47	75.01
13	1	1	0	19.18	78.12
14	0	0	0	16.42	85.36
15	1	−1	0	17.78	75.38
16	0	1	1	15.91	74.02
17	0	0	0	16.54	84.25

^a^The three levels (−1, 0 and 1) of factor X_1_ (extraction temperature) represented 70, 85 and 100 °C, respectively.

^b^The three levels (−1, 0 and 1) of factor X_2_ (extraction time) represented 1.0, 1.5 and 2.0 h, respectively.

^c^The three levels (−1, 0 and 1) of factor X_3_ (oxalic acid concentration) represented 0.1, 0.2 and 0.3%, respectively.

The generalized second-order polynomial model used in the response surface analysis was explained by Eq. (2) ^22^.

2 $$Y={{\rm{A}}}_{0}+{\sum }_{{\rm{i}}=1}{A}_{ii}{X}_{i}^{2}+\mathop{\sum }\limits_{{\rm{i}}=1}^{{\rm{n}}}\mathop{\sum }\limits_{{\rm{j}}={\rm{i}}+1}^{{\rm{n}}}{A}_{{\rm{ij}}}{X}_{{\rm{i}}}Xj$$

where *Y* is the response variables (extraction yield or uronic acid content); A_o_, A_i_, A_ii_ and A_ij_ are the regression coefficients for intercept, linear, quadratic and interaction terms, respectively; X_i_ and X_j_ represent the independent variables (i ≠ j). The models were used to evaluate the effect of each independent variable to the responses. The analysis of experimental design and calculation of predicted data were performed using Design Expert software 8.0.6 (Trial Version, State-Ease Inc., Minneapolis, MN, USA). According to analysis of variances (ANOVA which was applied to assess effects of studied variables, interactions and statistical significance of models) the fitness of the polynomial model equations were expressed by the coefficient of determination *R*^2^. Their statistical significances and significances of the regression coefficients were checked by *F*-test at a probability (*P*) of 0.001, 0.01 or 0.05^23^. The optimal extraction conditions were estimated through regression analysis and 3-D response surface plots. Then, three additional confirmation experiments were conducted to verify the validity of the statistical experimental strategies.

### Analysis methods

Uronic acid content was determined by the *m*-hydroxydiphenyl method^24^, using galacturonic acid as standard. Sugar composition analysis was performed by HPLC as previously described^25^. The molecular weight (*M*_w_) was estimated by high performance gel permeation chromatography (HPGPC) using a TSK-gel G-4000PW_XL_ column (7.8 × 300 mm, TOSOH, Japan) connected to a Shimadzu high performance liquid chromatography (HPLC) system^25^. The degree of esterification (DE) was determined by titration method according to the national standards of P.R.C. Ash content was determined at 500 °C for 8 h using a muffle furnace.

### Intrinsic viscosity

The extracted pectin LAHP and commercial LMP (CLMP) were dissolved in deionized water, then filtered through a 0.22 μm membrane and loaded in an ubbelohde capillary (diameter = 0.55 mm). Viscosity measurement was performed in a thermostatic water bath at 25 ± 0.1 °C. The initial pectin concentration was 1.0 mg/mL. And other concentrations were obtained by dilution sequentially with deionized water to 0.9, 0.8, 0.7, 0.6 and 0.5 mg/mL, respectively. Viscosity calculations were performed according to literatures^26,27^. The intrinsic viscosity [*η*] was estimated by extrapolation of Kraemer curves to “zero” concentration.

### Preparation of pectin gel and its textural properties

Pectin gel was prepared at a concentration of 10 g/L and sucrose content 10% as described previously^28^. Pectin and sucrose were dissolved in distilled water with mild agitation for 12 h at room temperature. After adjusted the pH to 4.0, the mixed liquid was added calcium chloride solution to obtain R = 0.58 (R = 2[Ca^2+^]/[COO^−^]) status. Then, the mixture was heated at 80 °C with agitation for 10 min immediately, cooled to room temperature and stored at 4 °C for 24 h. Before the texture analysis, the prepared gel was placed at room temperature for 0.5 h.

A Texture Analyser TA.XT Plus (Stable Micro Systems, UK) was used to determine the textural properties of the gel. The compression tests were performed using a cylindrical probe (5-mm radius, PC-0.5 R) as described previously^26^. A standard program was used to compress the gel by probe with 1 g original force at 0.1 mm/s and the puncture test was stopped when probe was penetrated into the gel 10 mm, after which the probe was withdrawn from the gel at 0.1 mm/s. Textural parameters of firmness, consistency, cohesiveness and viscosity index of pectin gel were obtained^29^. For gel penetration, the maximum positive force was taken as gel firmness and the positive area was recorded as consistency. The maximum negative force was taken as cohesiveness and the negative area was taken as viscosity index.

### Statistical analysis

All data were expressed as means ± standard deviation (S.D.). Statistical analyses were performed using Prism 5 Software. Comparisons between groups were performed using t tests or one-way analysis of variance (ANOVA) with Ducan’s range tests. Differences were considered significant when *p* < 0.05.

## Results and Discussions

### The results of single-factor experiments

#### Effect of extraction temperature on yield and uronic acid content

**Figure 1:**
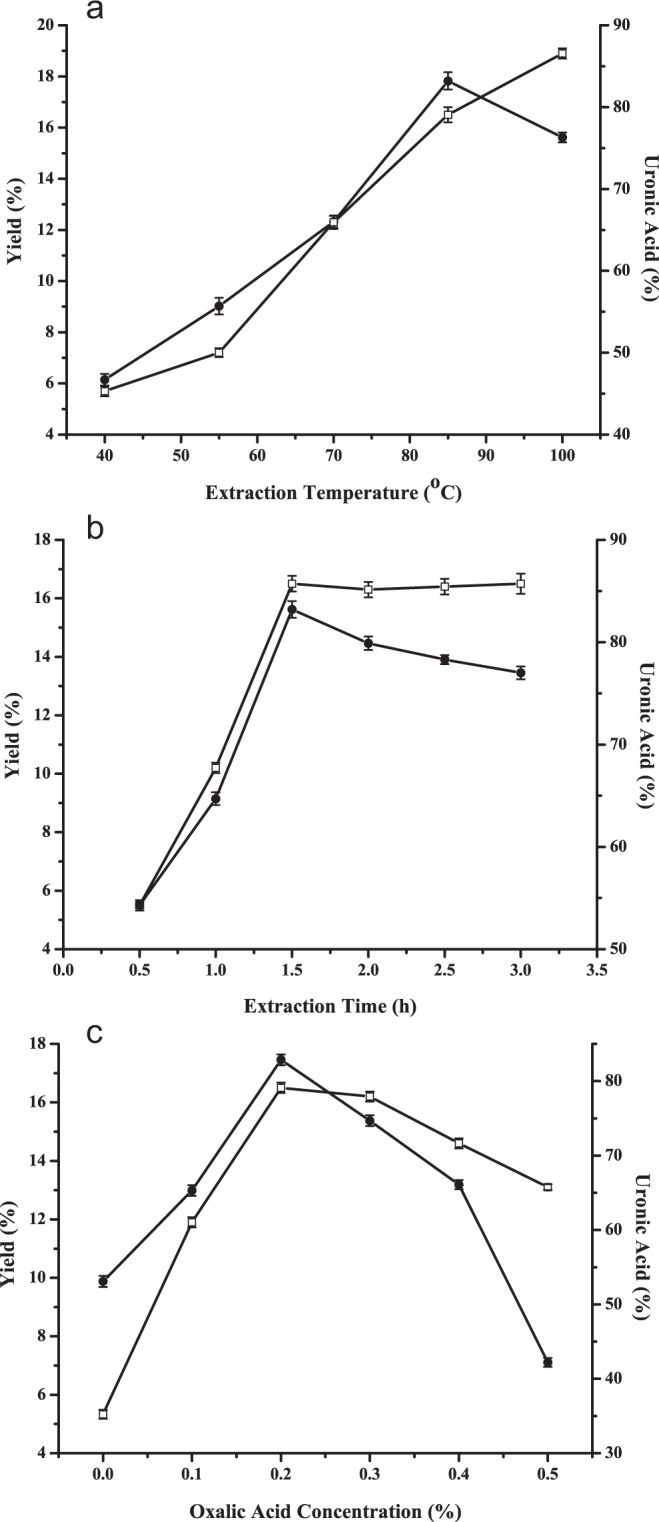
Effect of (**a**) extraction temperature, (**b**) extraction time, (**c**) oxalic acid concentration on the yield and uronic acid content of LAHP (-□- yield, -●- uronic acid content).

Extraction temperature was set at 40 °C, 55 °C, 70 °C, 85 °C and 100 °C to investigate its effect on extraction yield and uronic acid content of LAHP while extraction time was fixed at 1.5 h and oxalic acid concentration was fixed at 0.2%. As shown in Fig. 1a, there was an increasing trend in extraction yield of LAHP from 40 °C to 100 °C, which was in agreement with other reports on polysaccharides extracting^23,30^. The uronic acid content increased when extraction temperature increased from 40 °C to 85 °C, while decreased when temperature increased from 85 °C to 100 °C. The highest yield was at 100 °C but uronic acid content was decreased which may be due to the side reactions of GalA residues such as β-elimination and oxidation at high temperature^31^. Moreover, higher extraction temperature would increase more cost for industrial extraction process. Therefore, 85 °C was considered to be optimal temperature in the present experiment.

#### Effect of extraction time on yield and uronic acid content

Extraction time is another important factor that would influence the extraction efficiency because it is time consuming for LAHP to the release pectin. To study the effect of extraction time on yield and uronic acid content of LAHP, extraction process was carried out using the different time quantum of 0.5, 1.0, 1.5, 2.0, 2.5 and 3.0 h while other extraction parameters were fixed as follows: extraction temperature of 85 °C, oxalic acid concentration of 0.2%. Results in Fig. 1b showed that extraction yield and uronic acid content of LAHP increased rapidly as extraction time prolonged from 0.5 to 1.5 h. Although the extraction time was lengthened, extraction yield of LAHP had little differences and the uronic acid content had a slow decrease. It could be found that long extraction time will lead to the degradation of pectin. The results indicated that 1.5 h was the most favorable time for LAHP extracting.

#### Effect of oxalic acid concentration on yield and uronic acid content

The effect of oxalic acid concentration in extractant on yield and uronic acid content of LAHP was shown in Fig. 1c. The extraction was carried out with different oxalic acid concentrations when other extraction conditions were fixed as follows: extraction temperature of 85 °C, extraction time of 1.5 h. A considerable improvement of both extraction yield and uronic acid content was increased among the oxalic acid concentration from 0 to 0.2%. However, both of extraction yield and uronic acid content decreased when the concentration exceeded 0.2%, especially the uronic acid content. Oxalic acid as chelating agent could combine with Ca^2+^ in pectic acid to change the insoluble pectin into soluble pectin. Proper oxalic acid concentration in extractant was positive for pectin extraction. However, high oxalic acid concentration would decrease the pH of solution, which would cause the hydrolysis of pectin. The results suggested that oxalic acid concentration of 0.2% was the optimal conditions for extracting LAHP.

### Optimization of extraction condition by RSM

#### Extraction yield

The matrix of Box-Behnken experimental design and experimental results were presented in Table 1. It could be seen that there was a considerable variation in extraction yield of LAHP with different extraction conditions. By multiple regression analysis on the experimental data, the predicted response for the extraction yield of pectin could be obtained via the second-order polynomial equation in Eq. (3).

3 $$\begin{array}{rcl}{\rm{Yield}} & = & 16.58+3.86{{\rm{X}}}_{1}+0.93{{\rm{X}}}_{2}+0.42{{\rm{X}}}_{3}-0.14{{\rm{X}}}_{1}\,{{\rm{X}}}_{2}+0.19{{\rm{X}}}_{1}\,{{\rm{X}}}_{3}-0.17{{\rm{X}}}_{2}\,{{\rm{X}}}_{3}\\  &  & -\,1.28{{{\rm{X}}}_{1}}^{2}-0.85{{{\rm{X}}}_{2}}^{2}-1.09{{{\rm{X}}}_{3}}^{2}\end{array}$$

where X_1_, X_2_ and X_3_ were the coded values of the independent variables, extraction temperature, time and oxalic acid concentration.

The results were conducted by analysis of variance (ANOVA) as shown in Table 2. *F*-value (265.05) of model and the associated *p*-value (*p* <0.0001) indicated that the regression model was significant^32^. *F*-value (4.09) for the lack of fit was insignificant (*p*å 0.05) thereby was adequate for confirming the validity of the model^22^. The high value of *R*^2^ (0.9971) and adj-*R*^2^ (0.9933) indicated that the form of the model represented the actual relationship was well correlated between the response and independent variables^33^. At the same time, a low value (1.63) of coefficient of the variation (CV) clearly indicated a high precision and a good reliability of the experimental values^7^. The significance of each coefficient of Eq. (3) based on *p*-value was also listed in Table 2. The *p*-value was smaller and the corresponding coefficient was more significant. It could be seen from Table 2 that the linear coefficients (X_1_, X_2_ and X_3_) and quadratic term coefficients (X_1_^2^, X_2_^2^ and X_3_^2^) had significant differences with small *p*-values (*p* < 0.05) while other term coefficients were not significant.

**Table 2:** Results of ANOVA for extraction yield and uronic acid content of LAHP.

Source	Sum of squares	DF	Mean square	*F* value	*p*-value
**Yield (%)**					
Model	144.30	9	16.03	265.05	<0.0001
X_1_	118.97	1	118.97	1966.58	<0.0001
X_2_	6.85	1	6.85	113.15	<0.0001
X_3_	1.42	1	1.42	23.47	0.0019
X_1_ X_2_	0.081	1	0.081	1.34	0.2846
X_1_ X_3_	0.14	1	0.14	2.39	0.1663
X_2_ X_3_	0.11	1	0.11	1.86	0.2154
X_1_^2^	6.94	1	6.94	114.75	<0.0001
X_2_^2^	3.05	1	3.05	50.47	0.0002
X_3_^2^	5.04	1	5.04	83.30	<0.0001
Residual	0.42	7	0.060		
Lack of Fit	0.32	3	0.11	4.09	0.1035
Pure error	0.10	4	0.026		
Total	144.73	16			
R^2^ = 0.9971, adj-R^2^ = 0.9933, CV = 1.63					
**Uronic acid content (%)**					
Model	1529.35	9	169.93	113.34	<0.0001
X_1_	362.61	1	362.61	241.87	<0.0001
X_2_	75.22	1	75.22	50.17	0.0002
X_3_	34.40	1	34.40	22.95	0.0020
X_1_ X_2_	2.58	1	2.58	1.72	0.2313
X_1_ X_3_	1.22	1	1.22	0.81	0.3968
X_2_ X_3_	0.17	1	0.17	0.11	0.7475
X_1_^2^	298.30	1	298.30	198.97	<0.0001
X_2_^2^	150.54	1	150.54	100.42	<0.0001
X_3_^2^	501.13	1	501.13	334.26	<0.0001
Residual	10.49	7	1.50		
Lack of Fit	8.26	3	2.75	4.93	0.0787
Pure error	2.23	4	0.56		
Total	1539.84	16			
R^2^ = 0.9932, adj-R^2^ = 0.9844, CV = 1.68					

**Figure 2:**
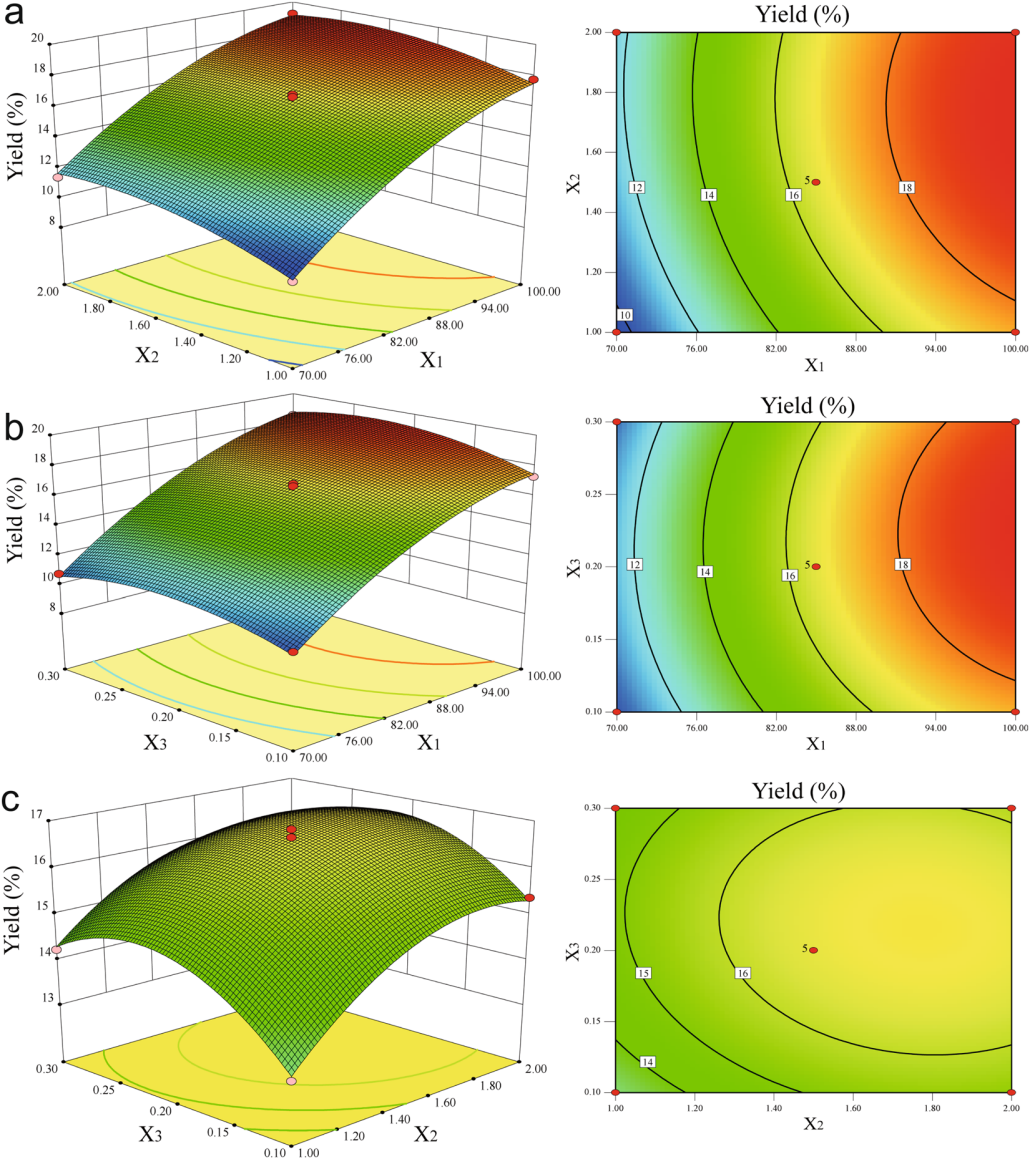
Response surface and contour plots showing the effect of the extraction temperature (X_1_), extraction time (X_2_) and oxalic acid concentration (X_3_) on the yield of LAHP. (**a**) Response surface and contour plots of yield as a function of X1 and X2. (**b**) Response surface and contour plots of yield as a function of X1 and X3. (**c**) Response surface and contour plots of yield as a function of X2 and X3.

The three-dimensional (3-D) response surface and contour plots were the graphical representations of the regression Eq. (3). It provided a method to visualize the relationship between responses and experimental levels of each variable parameters and the type of interactions between two test variables^34^. The results of extraction yield affected by extraction temperature, time and oxalic acid concentration were presented in Fig. 2a–c. In the response surface and contour plots, extraction yield was obtained along with two continuous variables while the other one was fixed constant at its zero level (center value of the testing ranges). It was clear that extraction yield was sensitive to minor alterations of the test variables. These figures showed that the extraction yield of pectin increased at first and then decreased with increasing of extraction time (Fig. 2a,c) and oxalic acid concentration (Fig. 2b,c), because the pectin would be degraded in long extraction time and high acid concentration by the side reaction of β-elimination and acid hydrolysis. Increasing extraction temperature leading to the increase of yield rapidly at first and then slowly indicated that higher extraction temperature was benefit to pectin extraction to some extent (Fig. 2a,b). According to the regression coefficients significance of the quadratic polynomial model (Table 2) and gradient of slope in 3-D and contour plots (Fig. 2), extraction temperature was the most significant factor to affect extraction yield followed by extraction time and oxalic acid concentration.

#### Uronic acid content of LAHP

According to the experimental results of Box-Behnken experimental design (Table 2), step-wise regression model of response surface for uronic acid content of LAHP was represented by the Eq. (4).

4 $$\begin{array}{rcl}{\rm{Uronic}}\,{\rm{acid}}\,{\rm{content}} & = & 84.82+6.73{{\rm{X}}}_{1}+3.07{{\rm{X}}}_{2}+2.07{{\rm{X}}}_{3}-0.80{{\rm{X}}}_{1}\,{{\rm{X}}}_{2}+0.55{{\rm{X}}}_{1}\\  &  & {{\rm{X}}}_{3}-0.21{{\rm{X}}}_{2}\,{{\rm{X}}}_{3}-8.42{{{\rm{X}}}_{1}}^{2}-5.98{{{\rm{X}}}_{2}}^{2}-10.91{{{\rm{X}}}_{3}}^{2}\end{array}$$

The results for the uronic acid content of LAHP were also analyzed by ANOVA as shown in Table 2. The high *F*-value of the model (113.34) and low *p*-value (*p* <0.0001) meant that the regression model was significant. High *R*^2^ (0.9932) and Adj-*R*^2^ (0.9844), low value of CV (1.68) and the insignificant *F*-value for the lack of fit (4.93) indicated that the mathematic model in Eq.(4) was adequate for predicting uronic acid content of LAHP under any combination of variables values. According to *p*-value of each coefficient of Eq. (4) in Table 2, the linear coefficients (X_1_, X_2_ and X_3_) and quadratic term coefficients (X_1_^2^, X_2_^2^ and X_3_^2^) had significant differences, but the other term coefficients were not significant.

**Figure 3:**
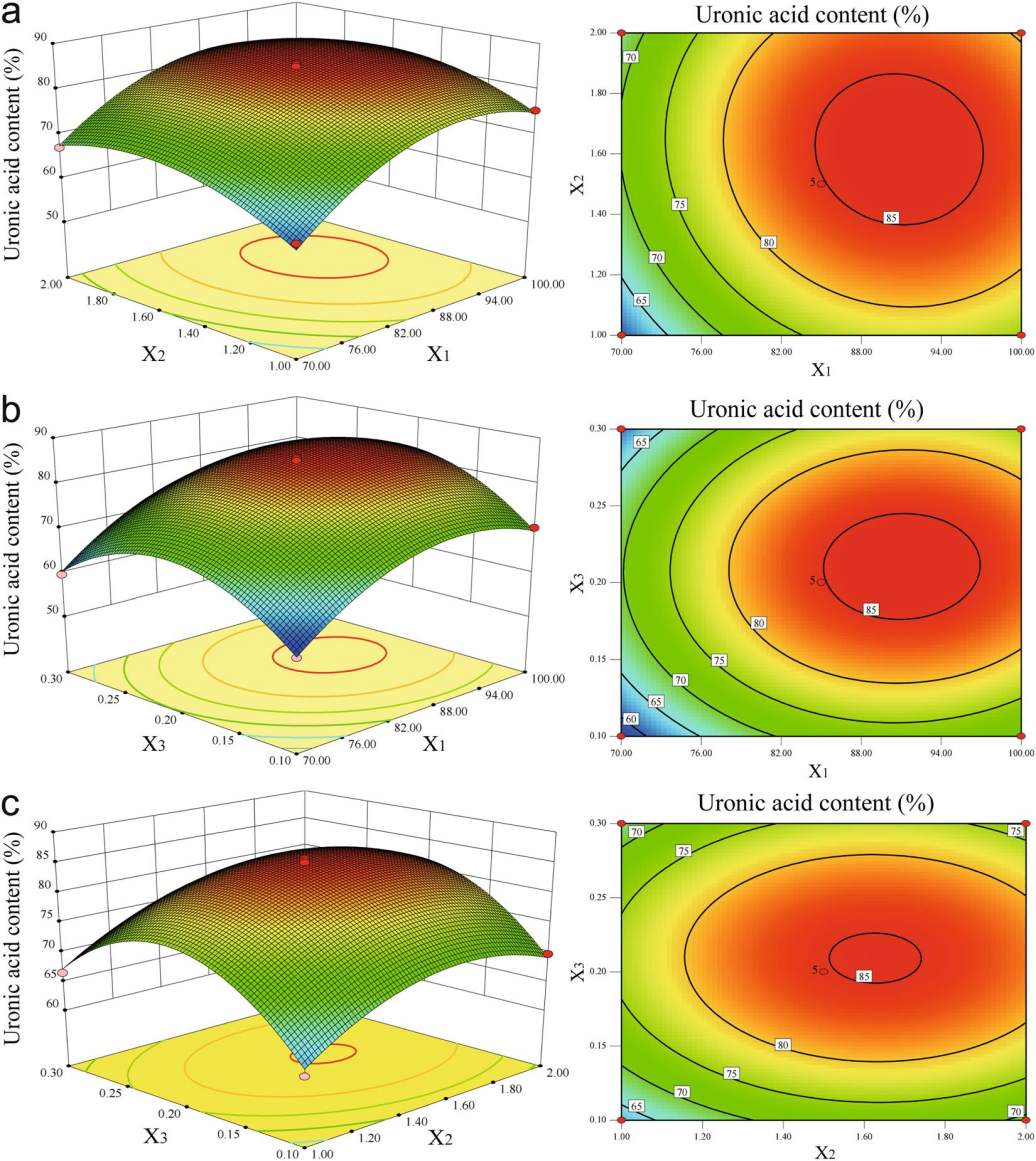
Response surface plots and contour plots showing the effect of the extraction temperature (X_1_), extraction time (X_2_) and oxalic acid concentration (X_3_) on the uronic acid content of LAHP. (**a**) Response surface and contour plots of the uronic acid content as a function of X1 and X2. (**b**) Response surface and contour plots of the uronic acid content as a function of X1 and X3. (**c**) Response surface and contour plots of the uronic acid content as a function of X2 and X3.

To visualize the relationship between independent variables and responses, the 3-D response surface and contour plots were generated for the models in function of two variables. The 3-D plots and contour plots in Fig. 3 showed the effects of extraction temperature, extraction time and oxalic acid concentration on the uronic acid content of LAHP. It was shown that uronic acid content increased rapidly at first and then decreased with increasing of extraction temperature (Fig. 3a,b), extraction time (Fig. 3a,c) and oxalic acid concentration (Fig. 3b,c). According to the regression coefficients significance of the quadratic polynomial model (Table 2) and gradient of slope the 3-D plots (Fig. 3), extraction temperature was the most significant factor to affect uronic acid content, then extraction time and oxalic acid concentration.

### Verification of predictive models

Optimum conditions for the extraction process were intended to obtain maximum extraction yield as well as higher uronic acid content. Based on the above findings, an optimization study was performed and the optimal conditions were determined as follows: extraction temperature of 96.05 °C, extraction time of 1.64 h and oxalic acid concentration of 0.21%. Triple validating experiments were conducted to confirm the prediction at a modified optimal condition in order to operate practically: extraction temperature of 96.0 °C, extraction time of 1.64 h and oxalic acid concentration of 0.21%. The extraction yield and uronic acid content were 18.83 ± 0.21% and 85.43 ± 2.9% respectively, which were approximately equal to the predicted yield (18.93%) and uronic acid content (85.56%) by the regression models.

### Chemical and Physical properties of LAHP

#### Chemical composition of LAHP

The chemical composition of sunflower head pectin extracted at the optimal conditions (LAHP) was shown in Table 3. The ash content of LAHP was 3.0%, which was lower than that extracted with sodium hexa-metaphosphate solutions^1,17^, but little higher than that of CLMP. The uronic acid contents of LAHP and CLMP were 86.3% and 76.8%, respectively, which coincident with the national standards of P.R.C (≥65%). Sugar composition determined by HPLC showed LAHP contained galacturonic acid (GalA, 84.6%) as the main component and trace amount of rhamnose (Rha, 6.5%), arabinose (Ara, 3.6%), glucose (Glc, 2.8%) and galactose (Gal, 2.5%). The ratio of Rha/GalA for LAHP was 0.077, which suggested the existance of large percentage of homogalacturonan (HG) and low percentage of type I rhamnogalacturonan (RG-I)^4,25^. It was noted that CLMP had the similar monosaccharides composition with some differences in the relative abundance of each monosaccharide (Table 3). The GalA content of LAHP (84.6%) was higher than that of CLMP (70.4%), while Glc content (2.8%) was lower than that of CLMP (18.9%), indicated that LAHP has rich pectin and less cellulose or starch-like glucan. LAHP was low methylated pectin with DE about 23.9%, lower than CLMP (33.8%). Furthermore, the DE of LAHP has little different with that extracted by 0.6% (w/v) sodium citrate^35^ (22.56%), but little higher than the DE of pectin extracted by sodium hexametaphosphate^1^ (11%). The *M*_w_ of LAHP (257.5 kDa) was lower than that of CLMP (463.4 kDa), due to the differences of plant sources, extraction methods and conditions.

**Table 3:** Chemical analysis of LAHP and CLMP.

Samples	Ash (%)	Uronic acid (%)	DE (%)	Sugar composition (%)					*M*w (kDa)
				GalA	Rha	Gal	Ara	Glc	
LAHP	3.0	86.3	23.9	84.6	6.5	2.5	3.6	2.8	257.5
CLMP	2.8	76.8*	33.8	70.4	2.9	4.1	0.7	18.9	463.4

#### Intrinsic viscosity of LAHP

**Figure 4:**
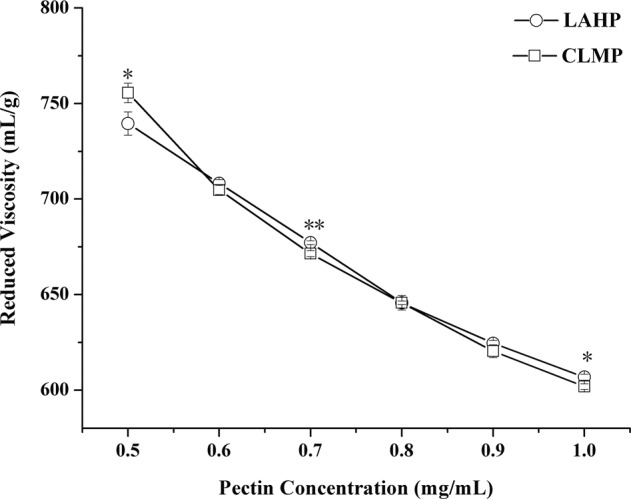
Huggins plot of LAHP and CLMP in deionized water (*p < 0.05, **p < 0.01).

Huggins plot of the reduced viscosity (*η*_red_) against the concentration of pectin aqueous solution was showed in Fig. 4. LAHP and CLMP had same *η*_red_ at initial concentration. With diluted of solution, *η*_red_ of LAHP and CLMP increased due to the increase of hydrodynamic volume which was caused by the electrostatic repulsions between dissociated carboxyl groups along pectin chains and the reduced steric hindrance of the pectin molecules in the low concentrated solutions^26^. Moreover, the two kind of pectin had similar intrinsic viscosities ([*η*]) of 866 mL/g and 890 mL/g, respectively. The [*η*] of LAHP was lower than that of sunflower head pectin SFHP^36^ and higher than that of sunflower head pectin KIM and LIN^1^. It had been reported that pectin with molecular weight <100,000 g/mol obeyed the Mark-Houwink relation of [*η*] = 9.55 × 10^−2^*M*_w_^α^ at 25 °C irrespective of the DE and sources of pectin^37^. In this study, LAHP and CLMP had the similar [*η*] and significant different *M*_w_. The reason was that [*η*] represented the volume of per unit mass that the polymer °Ccupied in solution. Therefore the sizes of [*η*] were dependent not only on the structures of pectin but also on the conformations which were mainly influenced by the charge distribution and the molecular interactions. This result can support the conclusion that the conformation of LAHP belonged to rod-like model and more stiff than CLMP, due to its higher GalA content and lower DE.

#### Textural properties of LAHP

Compared with CLMP gel, the firmness, consistency, cohesiveness and viscosity index of LAHP gel were significantly different (Table 4). Both of the two samples were low-methoxyl pectin forming gels in the presence of calcium ions. The gel properties were affected by GalA content, *M*_w_ and DE. Although *M*_w_ of LAHP was lower, the higher GalA content and lower DE had positive effect on its gel properties since they were benefit to calcium ion binding capacity and formation of egg-boxes. According to the gel textural properties comparison between LAHP and CLMP, it suggested that LAHP gel contained more stable features and might be instead of CLMP gel applied in the food industry.

**Table 4:** The comparison of the gel textural properties between LAHP and CLMP.

Sample	Firmness (g)	Consistency (g.s)	Cohesiveness (g)	Viscosity index (g.s)
LAHP	8.0 ± 0.2	713.8 ± 8.5	−3.00 ± 0.1	−129.3 ± 8.7
CLMP	10.1 ± 0.5**	869.9 ± 55.5**	−1.27 ± 0.1***	−32.9 ± 3.1***

**p < 0.01,***p < 0.001.

## Conclusions

In this work, natural low-methoxyl pectin extracted from sunflower heads with oxalic acid were investigated based on the Box–Behnken design statistical modeling. Considered the feasibility of experiment conditions, temperature of 96 °C, time of 1.64 and oxalic acid concentration of 0.21% were chosen to obtain the low methoxyl pectin LAHP. The extraction yield, uronic acid content, ash, DE, GalA content, molecular weight of LAHP were 18.83%, 85.43%, 3.0%, 23.9%, 84.6% and 257.5 kDa, respectively. Compared with commercial pectin CLMP, LAHP had lower *M*_w_, DE, neutral sugars contents, but higher uronic acid and ash contents, which might be owing to the more stiff rod-like conformation of LAHP. LAHP could form Ca^2+^-pectin gels with similar textural properties compared to CLMP. Therefore, it is a valid recycle use for waste resources of sunflowers and provides a new idea to explore the natural resources of low methoxyl pectin for food, cosmetic and pharmaceutical industries.
